# Measuring the positive potential value of birth in economic evaluation of perinatal interventions

**DOI:** 10.3389/fgwh.2025.1492231

**Published:** 2025-06-20

**Authors:** Meghan Bruce Kumar, Mabel Berrueta, Ezequiel García-Elorrio, Mahesh C. Puri, Lisa Hinton, Dorothy Oluoch, Cicely A. Marston

**Affiliations:** ^1^ Department of Nursing, Midwifery and Health, Northumbria University, Newcastle upon Tyne, United Kingdom; ^2^ Department of Health Systems and Research Ethics, KEMRI-Wellcome Trust Programme, Nairobi, Kenya; ^3^ Department of Maternal and Child Health Research, Institute for Clinical Effectiveness and Health Policy, Buenos Aires, Argentina; ^4^ Center for Research on Environment Health and Population Activities (CREHPA), Lalitpur, Nepal; ^5^ Nuffield Department of Primary Care Health Sciences, University of Oxford, Oxford, United Kingdom; ^6^ Department of Public Health, Environments and Society, London School of Hygiene and Tropical Medicine, London, United Kingdom

**Keywords:** health economics, maternal health, economic evaluation, strengths-based, well-being, perinatal, person-centred care

## Introduction

Giving birth is a time of both risk and possibility: what happens during childbirth can be life-changing and has life-long implications for mother, baby and wider family. We call for a fundamental rethinking of economic evaluation of perinatal health interventions that recognises this: one that centers birthing people's experiences and accounts for the positive potential value of birth in their lives, acknowledging that birth is not an illness (1, 2) from which the best possible outcome is a return to ‘baseline’. This reframing, in line with the salutogenic model of birth (3) challenges the typically employed ‘deficit model’ approach (one that focuses on measuring severe morbidities and mortality as the most important outcomes of perinatal health interventions) (4–7). We suggest instead assessing the potential well-being increase that would result from a positive, empowered, family- and person-centred birth experience alongside high-quality clinical care. In this commentary, we examine the well-being enhancing potential of a positive birth experience and the justification for health economists to include this in economic evaluations of perinatal health interventions.

From this point, at the request of the Frontiers in Global Women's Health Chief Editorial Team, we will refer to ‘women’ when discussing people giving birth. We acknowledge that not everyone giving birth identifies as a woman and that using this shorthand risks being read as ignoring transgender and non-binary people.

## Giving birth can improve well-being: what does this mean for the health economics of the peripartum period?

Economic evaluations in all health areas often focus on mortality and (severe) morbidity as the key outcomes of health interventions impacting quality of and length of life (8–10). Focusing on these ‘universal’ outcomes allows economists and policy makers to compare across health areas and interventions. In the perinatal period, using these outcomes means newborn well-being outweighs that of the parent(s) because of their much larger potential number of life years to be gained. Using these outcomes in this way drives a focus on both clinical quality of care and on newborn care as the focus of improvement with the highest potential return.

Although a woman's outcomes might be outweighed by their infant's, if a simple cumulative total approach to economic evaluation were used, the distribution of these outcomes is also important. In the global community, the right to high-quality reproductive healthcare is acknowledged (11), though often unmet. Among outcomes to be considered for women giving birth, 99.2% survive childbirth and the post-partum period globally today, though there are still many unnecessary deaths. As the recent WHO report states, from 2016 to 2020 three quarters of countries had stagnant or increasing maternal mortality rates (MMR), with a significant reduction in MMR in just 31 countries globally (12). As such, while tracking MMR remains critical to ensure sustained health impact, including in the Sustainable Development Goals (SDGs), simply focusing on maternal mortality (Goal 3.1) is no longer a sensitive marker of the impact of maternal and perinatal health improvement interventions in many settings (13–15). Indeed, after decades of progress focused on maternal mortality, there is a recognition that childbirth can and should be evaluated beyond merely maternal or perinatal survival and incorporate aspects of thriving and the potential for transformation (16–19).

The literature suggests that experience of maternity services affects utilization and timeliness of care seeking for future reproductive health needs, including early ANC (to identify high-risk pregnancies), appropriate imaging, skilled birth attendance and indeed safe abortion care (20, 21). As such, a poor experience for one person during one pregnancy can create a cascade of future decision-making for them and their neighbors/family/friends involving delayed or avoided care-seeking. By using approaches driven by quantitative, process-oriented metrics of clinical protocols that directly lead to observable mortality and morbidity outcomes, economists neglect or under-value patient experience aspects of quality of care (22), which are likely to be more context-specific yet are critical to family-centred care and longer-term mental and physical health (23, 24). There is strong evidence on the long-lasting impact of birth experience (25, 26) and potential long-term impacts on bonding and capacity of mother and baby to thrive and thus to contribute economic ‘value’ to society, beyond these intrinsically valuable capabilities (27).

Existing measures and scales are inadequate to this task, though beginning to acknowledge it in different ways. To date, these efforts have primarily focused on outcomes measures, and patient-reported outcome measures (PROMs) as a category can speak to some outcomes of birth experience—as differentiated from patient-reported experience measures, that might more directly engage with the process or experience of birth. There is very limited use of PROMs in maternity and childbirth as yet, though there is growing interest (28). For example, the Birth Satisfaction Scale is a specific and important scale that we include in our ongoing assessment of existing measures for understanding birth experience (29). However, we posit that satisfaction is an outcome and birth experience is a process, albeit one that is intrinsically valuable. In a systematic review of birth outcomes, the authors found an initial list of 135 positive outcomes and over ten times that amount of negative outcomes, supporting the claim that “…effectiveness of intrapartum interventions was measured against adverse outcomes rather than increases in measures of health and well-being” (3).

To measure change and perhaps kick start further “improvements” in quality of maternity care, a different metric to evaluate progress against is needed—this means using these broader outcomes as well as including patient-reported aspects instead of focusing narrowly on death and disability, as well as including patient-reported aspects (30, 31). What is currently under-explored are the links between experience and relevant capabilities—moving from the idea of confidence or satisfaction to the actualisation of those issues (for example, mother-baby bonding or fulfilment of relationships including with baby). In the next section, we propose a metric that would build on the steps that have been taken in development and refining the limited set of maternity PROM questions. It is possible to identify, measure and value outcomes including and derived from the birth experience, though there is limited literature in this area. The quality of care literature, both generally (23, 32, 33) and specific to the perinatal period (34), is very clear that there are both clinical aspects of quality and experiential aspects of quality that are equally important. Do economists believe that birth experiences do not matter or is there some other reason we are not counting them?

## A strengths-based approach to assessing the outcomes of birth

Negative birth experiences are a big problem: about 130 million people give birth every year and estimates of the incidence of trauma in childbirth range from three to 33 percent of birthing women in different settings using different methodologies (35–37)—a great deal of trauma no matter how it is measured. Disrespect and abuse in maternity care, which could be generically termed negative or poor-quality patient experiences, are well-documented globally and often sources of psychological trauma (21, 38–41). Trauma-informed research and practice suggests that strengths-based approaches and empowerment are critical to avoiding trauma or retraumatisation (42, 43).

Why is it that these negative practices persist? Why are global investments in addressing deficits in maternal health not making the expected gains? Perhaps because we are focusing on a deficit model in an over-worked/burdened health system. Evidence from other health areas suggests that using positive deviants (44), exemplars (45) or strengths-based approaches can be a means of effectively changing culture, inspiring change in a health system made up of individual providers who are often under-resourced and under-supported. For example, a study looking at provider-to-provider teleconsultations in Kenya found benefits to the system (communication, teamwork) as well as to the individual providers (confidence, capacity) that led to the positive impacts of improved responsiveness (experience quality) and more accurate diagnoses (clinical quality) (46, 47). Strengths-based work can also learn from drivers of resilience for individuals, families and communities that lead to positive social and health outcomes, as in this adult social care example from the UK (48); an organising framework suggests considering strengths that are individual, interactional, and contextual (49).

Birth can be a well-being enhancing event for mother and baby in the short- and long-term and sets them up for healthy bonding and early development. In measuring the outcomes of birth, it is important to consider that we are evaluating something other than injury or illness and therefore has potentially *positive* impact on health and well-being. If we evaluated interventions in this period by measuring deviation from an ‘expected’ positive/transformative/well-being enhancing experience, this would change our expectations for the responsiveness of the health system. A strengths-based approach may also highlight pre-existing inequities in health and well-being, as it would measure utility gaps to a gold standard. This approach explicitly centres equity or differences in attainment of well-being between population sub-groups by incorporating pre-birth deficits in well-being into the metric—so one might see, for example, Black women in the UK having both a pre-birth well-being level that was lower than other racial groups and having less positive birth experiences. As such, the cumulative utility gap would look larger against this gold standard of attainment and thus would guide investment to these high-return population sub-groups or communities.

## A metric for positive potential value of birth

What would it look like for economists to centre women's positive birth experiences by comparing intervention impacts to a gold standard of birth? It would require a metric for the ‘positive potential value’ (PPV) of birth to measure the possibility of an empowering, positive birth experience that can improve well-being.

**Figure 1:**
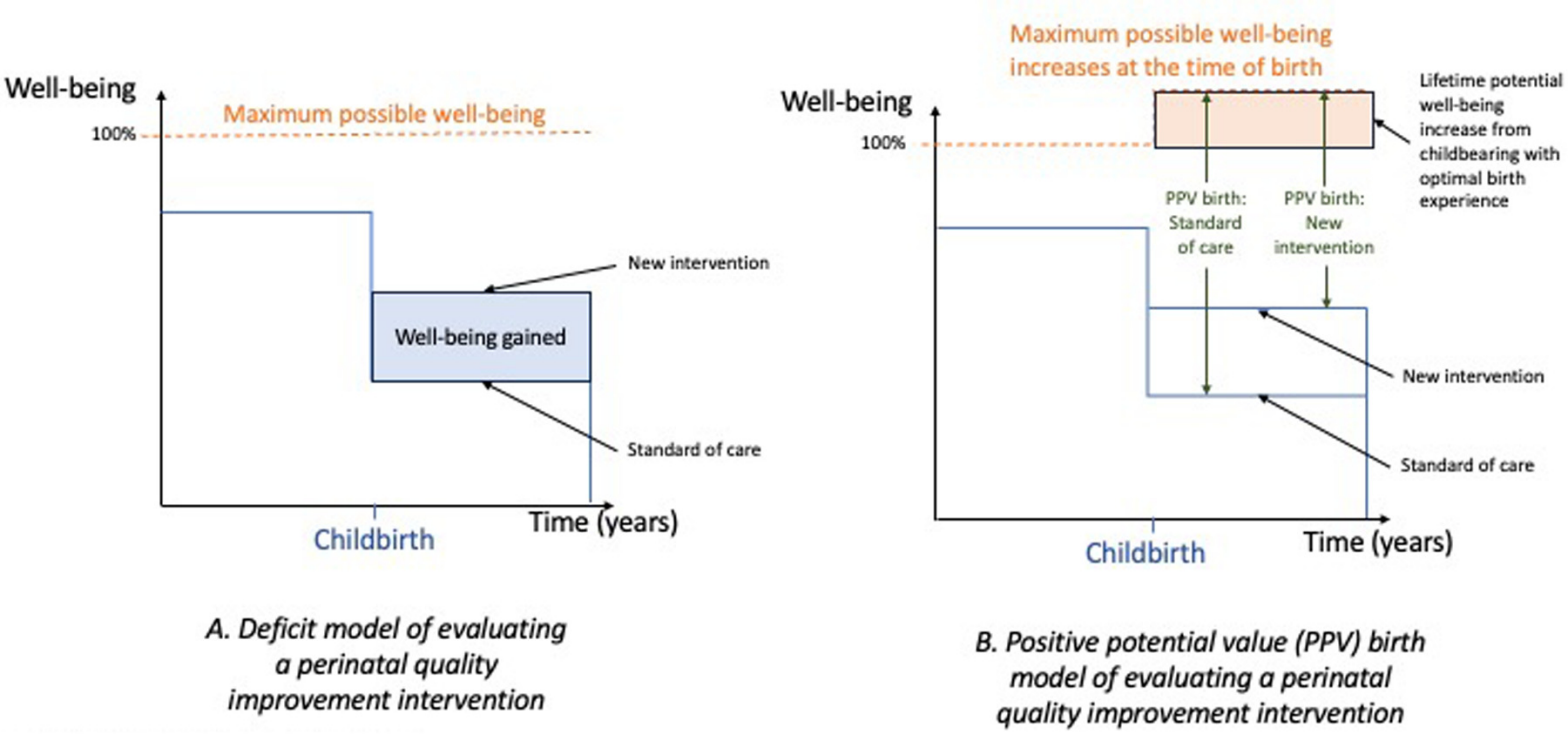
Defining the PPV birth metric. **(A)** A traditional pre/post measure of well-being gained under comparison of intervention and comparator is measured in the blue box (increase in well-being times years experienced). **(B)** The potential increase in the well-being frontier at the time of birth is shown in the shift in the orange dotted line. The PPV birth point measure is compared under the two perinatal interventions using green arrows (smaller values are better as the gap between the maximum and actual well-being is measured).

In Figure 1, we illustrate how pre-birth inequities in well-being would be captured in a gold standard PPV birth metric. Specifically, we use a parallel with traditional health economic evaluation. Economists look at full health as 1 or 100% and measure any deficit from that and the time incurred. In contrast in the PPV birth measure, if the individual's level of well-being before birth was low, even a maximum positive increase due to birth experience could only bring them up to a certain level of well-being post birth. A scale that measures capabilities is not incremental and therefore would incorporate the pre-birth level of well-being (or rather, the deficiencies in this due to individual characteristics including health and structural barriers). Using theory-driven evaluation approaches that look at the context and mechanisms of change to assess transferability of the findings are critical to making meaningful comparisons in other sites.

Building on work of the International Consortium for Health Outcomes Measurement (28) and learning from systematic review of patient-reported measures for childbirth (30) that acknowledges existing patient-reported maternity metrics are of poor quality/insufficiently validated, the PPV birth metric would provide an opportunity to collect routine data on patient experience, to augment the existing quantitative indicators of clinical processes and outcomes routinely collected through health management information systems. The PPV metric could be deployed directly at facility level to provide routine data on individual and facility performance management for quality improvement. Additionally, economists could improve the comprehensiveness of outcomes used in economic evaluation of maternal and perinatal interventions to incorporate aspects of positive experience.

There are a range of economic theories that could be applied to assessing the full PPV of birth. For example, applications of Sen's ‘capabilities approach’ (27) explores broader outcomes than health-related quality of life and freedoms to realise an individual's potential, including existing measures specific to women (50, 51). Prior economic evaluations have drawn on Sen's approach with a focus on specific time periods in the life course (e.g., palliative care, childhood) (52–54) or for specific health areas (mental illnesses) (55, 56) to elucidate what capabilities to achieve well-being might be, as understand by both patients and society at large.

Value-based care is a move towards improving patient experience by this by aligning metrics by which providers are evaluated to outcomes of interest to the patients and their families (28). Our approach is person-centred, like value-based care, but differs in two major aspects: first, that outcomes of interest to the patient are the focus of value-based care where we focus on experience and perhaps processes of care. Second, taking a strengths-based approach that views positive experience as attainable is another major difference from common ‘name and shame’ approaches to performance management.

**Figure 2:**
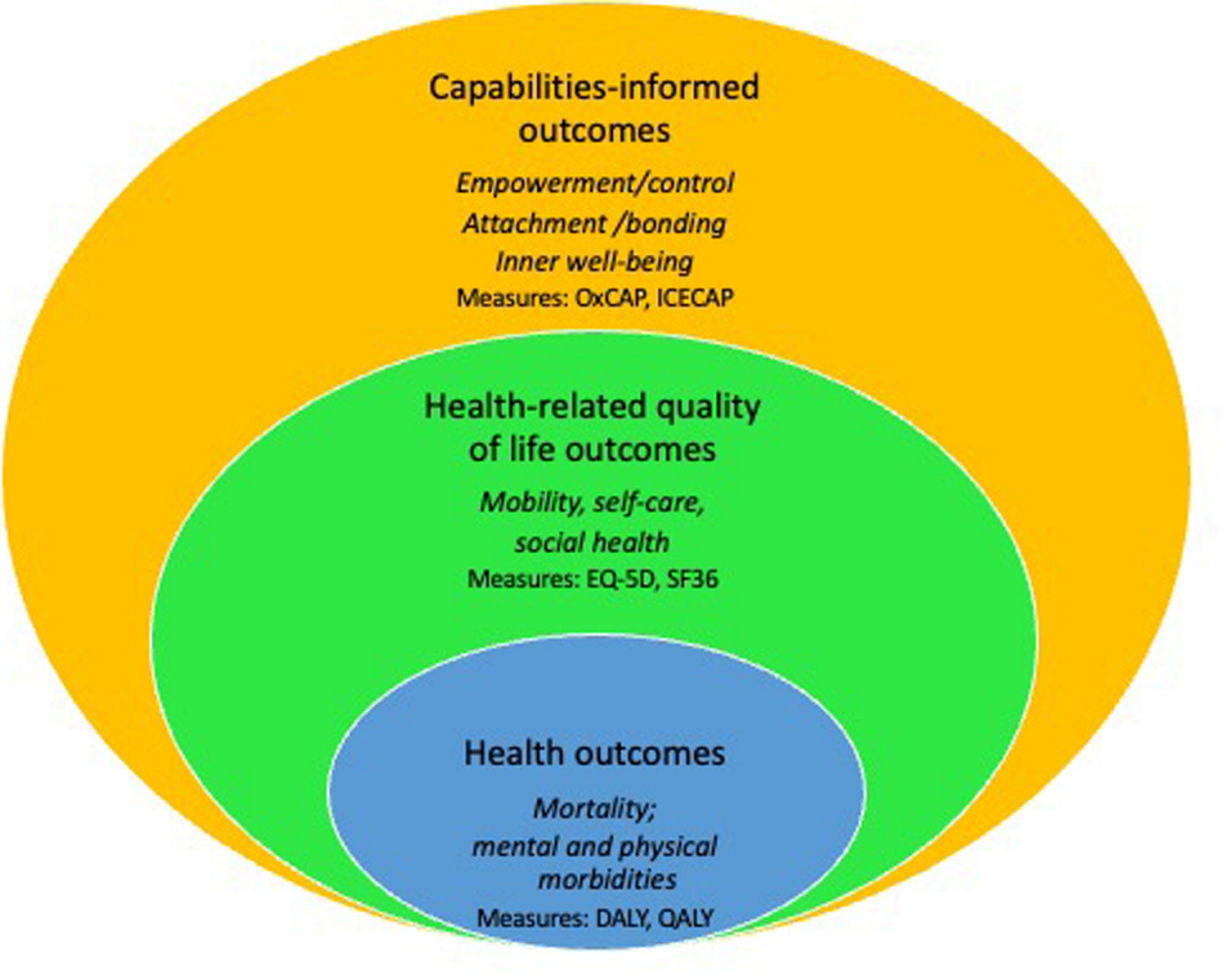
Categories of outcomes used in economic evaluation of perinatal interventions.

By centering experience in maternity care and focusing on the gold standard of positive, well-being enhancing aspects of birth experience as illustrated by the yellow circle of Figure 2, health systems can learn from strengths-based approaches to prioritize family-centred perinatal care that moves beyond valuing only health-related quality of life outcomes (57). Focusing on positive potential birth experience would allow those giving birth and evaluators to describe ‘what is possible’ in the context of maternity care under different system conditions and structures. Looking specifically at individuals’ experiences and specific facilities’ performance through the PPV metric provides a patient-centred way of exploring influence of context on intersectional vulnerabilities and experiences of birth and identifying opportunities for quality improvement in the health service provision/health system. For example, in a survey of maternal mortality and morbidity in Nepal, we found increased vulnerability and risk of inappropriate care (absence of regular service, long waiting hours, health facilities as being cold, dilapidated and lack of human resources) among socio-economically disadvantaged service users (58).

Yet there is an obvious tension or even conflict between metrics for quality and intersectionality considerations that shape experience. Across healthcare services, there remains a dearth of routine data on patient experiences—there are gold standards for clinical quality but not experiential quality (59). This tension is unresolved, but that does not imply it cannot be improved upon. For example, in the WHO quality of care framework for maternal and newborn health under the aspect focusing on experience, they include aspects such as: effective communication, respect and dignity, and emotional support (60). Accompaniment by a birth companion of one's choice is also a clear factor underpinning positive birth experiences (61, 62). It is not clear that these will materialise the same way for different individuals or be weighted the same way across contexts, but it is likely that these are universal categories of factors that will be present to ensure/enable a positive experience.

We need to work in engaged, participatory ways (63–66) with mothers, families and communities to define the gold standard of birth experience and how this varies with individual values and preferences and contextual norms and constraints. One approach would be to develop a richer understanding of women's positive or negative experiences through collecting narratives (22). How these deviate from the gold standard of a well-being enhancing birth could be a starting point to build an understanding of what high quality maternity care looks like to women and thereby examine quality variations in experience (22, 67). The subsequent development of a PPV of birth metric would be a first step towards focus on investing to achieve positive birth experiences with an explicit focus on the equity implications of current and future investments in maternal and perinatal health.

## Conclusion

In summary, economic evaluation of perinatal interventions using a strengths-based approach inclusive of birth experience and related outcomes can change decisions about what is a good investment at all levels of perinatal care. This approach will move us toward a world of more equitable access to high-quality care encompassing both clinical and experiential aspects of quality. Utilizing this new approach will help decision makers to prioritise resources in a way that reflects women's full potential for health and well-being.
